# Effect of Socioeconomic Status on Weight Change Patterns in Adolescents

**Published:** 2008-12-15

**Authors:** Nancy E. Sherwood, Melanie Wall, Dianne Neumark-Sztainer, Mary Story

**Affiliations:** HealthPartners Research Foundation. Dr Sherwood is also affiliated with the University of Minnesota, Minneapolis, Minnesota; University of Minnesota, Minneapolis, Minnesota; University of Minnesota, Minneapolis, Minnesota; University of Minnesota, Minneapolis, Minnesota

## Abstract

**Introduction:**

Although socioeconomic differences in prevalence of obesity are well documented, whether patterns of weight gain during key periods of growth and development differ among youth from different socioeconomic backgrounds is unknown. This study examines socioeconomic disparities in overweight status and 5-year weight gain among adolescents.

**Methods:**

Project EAT (Eating Among Teens)-II followed a socioeconomically and ethnically diverse sample of 2,516 adolescents from 1999 through 2004. Mixed-model regression analyses examined longitudinal trends in overweight status as a function of socioeconomic status (SES).

**Results:**

Girls and boys in the low-SES category were more likely to be overweight than were those in the high-SES category. Boys in the high-SES category showed a significant decrease (*P* = .006) in overweight prevalence between 1999 and 2004, whereas boys in the low- and middle-SES categories showed no significant change. Girls in the low-SES category showed a significant 5-year increase (*P* = .004) in overweight prevalence compared with a stable prevalence of overweight among girls in the middle- and high-SES categories.

**Conclusion:**

Our data show continued and, in some cases, increasing socioeconomic disparities in risk for overweight. Youth from low-SES backgrounds are at increased risk for overweight and are more likely to remain overweight (boys) or become overweight (girls). Designing obesity prevention and treatment interventions that reach and address the unique needs of youth and families from less-advantaged socioeconomic backgrounds is a public health priority.

## Introduction

Although the prevalence of overweight in adolescents has increased dramatically during the past several decades among all ethnic and socioeconomic status (SES) groups, considerable data suggest that risk for adolescent obesity is higher for certain racial/ethnic groups and is inversely associated with SES (1). Recent data, however, have raised the question of whether disparities in the prevalence of overweight among adolescents have been narrowing or widening (2-4).

To date, most studies examining SES disparities in weight have focused on secular trends (2,3). Although differences in obesity by SES are well documented, we do not know whether patterns of weight gain differ during key periods of growth and development among youth from different SES backgrounds. Longitudinal trends in overweight in adolescents have been examined as a function of race/ethnicity (5), but few studies focus on potential SES disparities in patterns of weight gain over time. A better understanding of associations between SES and race/ethnicity and changes in overweight status over time is needed to identify which subgroups of children are at particularly high risk of becoming and remaining overweight. Such information is critical for developing tailored obesity prevention and treatment interventions that will best meet the needs of high-risk groups.

This study examines longitudinal changes in weight status in a large, socioeconomically and ethnically diverse sample of adolescent boys and girls during a 5-year period. Specifically, this study sought to answer whether SES disparities in overweight status and weight gain persist over time among adolescents and whether these patterns are different for boys and girls.

## Methods

### Study sample

Project EAT (Eating Among Teens)-II is a follow-up study of Project EAT-I, a study of the determinants of dietary intake and weight status in adolescents (6-8). In Project EAT-I, 4,746 middle and high school students in 31 Minnesota schools completed in-class surveys and anthropometric measures during the 1998-1999 academic year. Participants were surveyed again by mail in Project EAT-II, 5 years later (2003-2004), as the younger cohort progressed from early adolescence to middle adolescence, and the older cohort progressed from middle adolescence to late adolescence/young adulthood. Twenty-three percent (n = 1,074) of participants were lost to follow-up for several reasons, including missing contact information at EAT-I (n = 411) and no address located at follow-up (n = 591). The remaining 3,672 participants were contacted by mail; 2,516 participants completed surveys, which represented 53% of the original cohort and 68% of participants who could be contacted for Project EAT-II. The study sample for these analyses included 1,074 boys and 1,334 girls who completed surveys for both EAT-I and EAT-II and who did not have missing data for the SES variable (108 were missing data). A greater proportion of the EAT-II survey respondents were female, white, and of higher SES than were EAT-I respondents; therefore, data were weighted by using the response propensity method. The University of Minnesota's institutional review board human subjects committee approved all study protocols.

### Measures

For EAT-I, weight and height were measured and self-reported, and body mass index (BMI) was computed. For EAT-II, self-reported weight and height data were collected in the mail survey. Participants were asked to report, "How tall are you?" in feet and inches and "How much do you weigh?" in pounds. The trend data we report use the self-reported figures from EAT-I, although if self-reported BMI was not available (n = 117), items were imputed based on baseline-measured BMI, age, race, and SES within sex. The measured and self-reported BMI in EAT-I were highly correlated (*r* = .85 for girls, *r* = .89 for boys). We classified overweight as BMI more than the 85th percentile for sex and age according to the first National Health and Nutrition Examination Survey because it provides values from childhood to adulthood, and in Project EAT-II adolescents are followed through late adolescence/early adulthood (9,10).

Sex, age, race/ethnicity, and SES were based on self-report in EAT-I. The primary determinant of SES was parents' education, defined by the higher level of either parent (11). Other variables used to assess SES included family eligibility for public assistance (yes, no, don't know), eligibility for free or reduced-cost school meals (yes, no, don't know), and maternal and paternal employment status (full-time, part-time, not working, don't know). We developed an algorithm by using classification and regression trees (12), in which a missing variable is replaced by a correlated surrogate variable. Algorithmic classification scores were reduced by 2 levels if the family received public assistance and by 1 if the child was eligible for free or reduced-cost school meals or had 2 unemployed parents. On the final classification tree, only 4% had no data on which to assign a highest level of parents' education and SES. Reports of SES were validated in a substudy of 902 parents, 876 of whom provided information on education, employment status, and income. The Spearman correlation for the association between parents' and adolescents' reports of SES was .67, and the weighted k (Cohen) was .79. The 5-level SES variable (low, middle-low, middle, middle-high, high) was trichotomized for these analyses (low/middle-low [low], middle, and middle-high/high [high]).

### Statistical analysis

Because attrition in the sample did not occur completely at random, the data were weighted by using the response propensity method (13) in which the inverse of the estimated probability that an individual responded to EAT-II was used as the weight. These procedures are described in detail elsewhere (8). The weighted sample has a similar demographic makeup to that of the original Project EAT-I sample.

Analyses examined whether longitudinal trends in the prevalence of overweight vary as a function of SES. Longitudinal trends were estimated and tested by using participants who had self-reported BMI data available in both surveys. We used mixed model regressions that included a main effect for year (1999 or 2004), cohort (younger or older), SES (low, middle, high), and an SES-by-year interaction along with a random effect for individuals to account for longitudinal correlation to estimate and test difference of prevalence of overweight across time, both within and across SES categories. Analyses were stratified by sex and adjusted for race/ethnicity and cohort. SAS version 9.1 (SAS Institute Inc, Cary, North Carolina) was used for all analyses.

## Results

The sample was relatively evenly distributed across the 3 SES categories (Table). Approximately half of the sample was white; however, white adolescents were overrepresented in the high-SES category. Across the sample, the prevalence of overweight (approximately 26%) did not significantly change from 1999 to 2004 (*P* = .71). However, when change in overweight status over time was broken down by SES, differential change patterns emerged; the prevalence of overweight increased for adolescents in the low-SES group from 29.8% to 33.1% (*P* < .001) and decreased for adolescents in the high-SES group from 23.1% to 19.2% (*P* < .01).

In both 1999 and 2004, a larger proportion of boys in the low-SES category were overweight than were boys in the high-SES category (1999, *P* = .02; 2004, *P* < .001) (Figure). In 1999, more boys in the low-SES category than in the middle-SES category were overweight (*P* = .007). Although the prevalence of overweight among boys did not show an overall significant longitudinal increase from 1999 to 2004 (*P* = .33), changes in overweight prevalence during this period varied as a function of SES. In particular, the SES-by-year interaction was significant (*P* = .03), and boys in the low- and middle-SES categories showed no significant change in overweight prevalence from 1999 to 2004, compared with boys in the high-SES category, who showed a significant decrease (*P* = .006). No significant interaction with cohort was observed, which suggests that these results did not vary between the younger and older cohorts. Race/ethnicity was not a significant predictor of overweight prevalence among boys after adjusting for SES. Additionally, the interaction between race/ethnicity and time was not significant, which suggests that changes in overweight prevalence over time did not vary by race/ethnicity among boys after accounting for SES.

**Figure .**
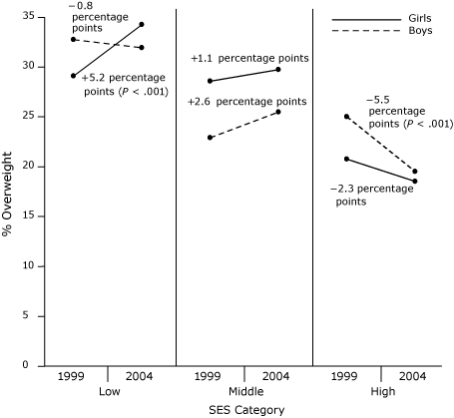
Changes in the prevalence of overweight by sex and socioeconomic status (SES) category among adolescents in the Project EAT (Eating Among Teens) study (1998-1999 to 2003-2004).

In 1999 and 2004, a larger proportion of girls in the low-SES category were overweight than were high-SES girls (1999, *P* = .005; 2004, *P* < .001). As with boys, the overall prevalence of overweight did not change from 1999 to 2004 (*P* = .22). However, a significant SES-by-year interaction was observed (*P* = .02). Girls in the low-SES category showed a significant increase (*P* = .004) in the prevalence of overweight, compared with the relatively stable prevalence of overweight among girls in the middle- and high-SES categories. Race/ethnicity remained a significant predictor of overweight prevalence after adjusting for SES, which suggests that race/ethnicity and SES were independently associated with prevalence of overweight in girls. However, the interaction between race/ethnicity and time was not significant, which suggests that changes in overweight prevalence over time did not vary by race/ethnicity after accounting for the role of SES.

## Discussion

Our findings confirm SES disparities in the prevalence of overweight; boys and girls in the low-SES category are at highest risk. Moreover, SES is associated with different patterns of weight change over time for adolescent boys and girls. Although boys of low and middle SES showed a consistent, relatively high prevalence of overweight during the 5-year study period, boys in the high-SES category showed a significant decrease in the prevalence of overweight. Girls in the low-SES category showed a significant increase in the prevalence overweight during the 5-year period, in contrast with the relatively stable prevalence of overweight among girls in the middle- and high-SES categories, further widening the SES disparities in the prevalence of overweight.

These data from Project EAT contribute to the growing complex and occasionally contradictory literature on relationships among weight status, SES, race/ethnicity, and sex. Two recent studies have suggested that SES disparities in overweight status have been narrowing. Between 1988-1994 and 1999-2002, the ratio in the prevalence of overweight between high- and low-SES adolescent boys and girls decreased significantly (3). Similarly, the Add Health Study (14) found ethnic disparities in the prevalence of overweight by SES. In contrast, analyses of the 4 cross-sectional National Health and Nutrition Examination Survey (NHANES) surveys from 1971-1974 to 1999-2004 showed that secular trends in overweight status varied by SES; older adolescents from lower SES backgrounds showed the least favorable pattern (2). Our longitudinal data support the finding of continued and, in some cases, increasing SES disparities in risk for overweight. Specifically, low-SES youth, and in some cases middle-SES youth, are not only at increased risk for overweight but are also more likely to stay overweight (boys) or become overweight (girls) as they get older.

The Add Health Study (14) also showed variation by sex, with a clear inverse association between SES and obesity in girls but not boys. Recent analysis of NHANES 1999-2002 data also showed no consistent association between SES and overweight for boys, but low-SES adolescent girls had a much higher prevalence than did their medium- and high-SES counterparts (3). However, this difference was due mainly to the strong inverse association between SES and overweight among white girls. In this study, high-SES black girls were at increased risk compared with their lower-SES counterparts (3). Further exploration of cultural, behavioral, psychological, and biological factors that may be associated with these complex relationships is warranted.

Our study has a number of strengths, including a large and diverse sample in terms of SES and race/ethnicity, a multifactorial measure of SES, and the ability to examine 5-year longitudinal trends. However, some limitations also need to be taken into account when interpreting the findings. In spite of multiple attempts to reach the original study participants, attrition was high, but use of a weighted sample reduces this concern. Although Project EAT included a diverse sample, some cell sizes for race/ethnicity were small, which decreased our ability to examine certain racial/ethnic and SES weight trends. Additionally, sexual maturation was not assessed; therefore, relationships between the timing of pubertal and BMI changes and variations in these relationships by sex and race/ethnicity could not be examined. Finally, BMI values were based on self-report, although measured and self-reported BMI were highly correlated in EAT-I.

Our findings underscore the need for obesity prevention and treatment for youth in general, given the overall high prevalence rates, but specifically for interventions tailored for low-SES groups. The school environment is an excellent place to reach out to low-SES youth since most children attend school, and barriers to engaging in physical activity and nutrition interventions, such as cost and transportation, are markedly reduced. Designing obesity prevention and treatment interventions that reach and address the unique needs of low-SES youth is a public health priority because of the increased prevalence of overweight and the high likelihood that these adolescents will remain overweight as adults.

## Figures and Tables

**Table.** Characteristics of the Project EAT Study Sample, by SES, at Baseline (1998-1999) and Change in Weight Status from 1999 through 2004^a^

**Characteristic**	Total Sample (N = 2,408)	Low SES (n = 884)	Middle SES (n = 642)	High SES (n = 882)	*P* Value^b^
**Sex**					
Boys, % (n)	44.6 (1,074)	40.1 (355)	46.7 (300)	47.5 (419)	.002
Girls, % (n)	55.4 (1,334)	59.9 (529)	53.3 (342)	52.5 (463)	
**Mean age at EAT-I (SD), y**	15.0 (1.6)	15.1 (1.8)	14.8 (1.7)	14.9 (1.4)	.001
**Race/ethnicity**					
White, % (n)	49.8 (1,185)	27.9 (243)	48.7 (310)	72.4 (632)	<.001
African American, % (n)	18.7 (444)	21.9 (191)	23.2 (146)	12.1 (106)	
Hispanic, % (n)	5.8 (139)	9.0 (76)	5.9 (37)	2.6 (23)	
Asian, % (n)	18.6 (443)	32.5 (283)	14.4 (92)	7.8 (68)	
Native American, % (n)	3.4 (80)	3.7 (32)	4.1 (26)	2.5 (22)	
Mixed or other race, % (n)	3.7 (89)	5.0 (44)	3.6 (23)	2.6 (23)	
**Weight status**					
1999					
Mean BMI (SD), kg/m^2^	22.44 (4.57)	23.03 (5.34)	22.34 (4.94)	21.92 (3.66)	.005
Overweight, % (n)	26.1 (613)	29.8 (256)	25.3 (157)	23.1 (200)	<.001
2004					
Mean BMI (SD), kg/m^2^	24.32 (5.01)	25.14 (6.25)	24.24 (5.11)	23.57 (3.78)	<.001
Overweight, % (n)	26.5 (623)	33.1 (283)	27.6 (172)	19.2 (167)	<.001
Change in overweight, 1999-2004, percentage points	+0.4	+3.3	+2.3	−3.9	<.001

Abbreviations: EAT, Eating Among Teens; SES, socioeconomic status; BMI, body mass index.

a The sample size for different variables may vary from the total sample size because of missing responses. Percentages may not add up to 100% because of weighting.

b The *P* value is for the associated test of difference across SES categories.
